# The effects of plant stanol ester consumption on arterial stiffness and endothelial function in adults: a randomised controlled clinical trial

**DOI:** 10.1186/1471-2261-13-50

**Published:** 2013-07-10

**Authors:** Helena Gylling, Janne Halonen, Harri Lindholm, Jussi Konttinen, Piia Simonen, Markku J Nissinen, Aslak Savolainen, Airi Talvi, Maarit Hallikainen

**Affiliations:** 1 Department of Medicine, Division of Internal Medicine, University of Helsinki, Helsinki, Finland; 2 Department of Clinical Nutrition, Institute of Public Health and Clinical Nutrition, University of Eastern Finland, Kuopio, Finland; 3 Finnish Institute of Occupational Health, Helsinki, Finland; 4 Heart and Lung Center, Helsinki University Central Hospital, Helsinki, Finland; 5 Department of Medicine, Division of Gastroenterology, University of Helsinki, Helsinki, Finland; 6 Finnish Broadcasting Company and Department of Public Health, University of Helsinki, Helsinki, Finland; 7 SOK Corporation, Helsinki, Finland; 8 Biomedicum Helsinki C 4 22, P.O. BOX 700, FIN-00029, HUS, Helsinki, Finland

**Keywords:** Arterial stiffness, Endothelial function, Cardio-ankle vascular index, Reactive hyperemia index, Augmentation index, Plant stanol ester, LDL cholesterol, Coronary artery disease

## Abstract

**Background:**

The hypocholesterolemic effect of plant stanol ester consumption has been studied extensively, but its effect on cardiovascular health has been less frequently investigated. We studied the effects of plant stanol esters (staest) on arterial stiffness and endothelial function in adults without lipid medication.

**Methods:**

Ninety-two asymptomatic subjects, 35 men and 57 women, mean age of 50.8±1.0 years (SEM) were recruited from different commercial companies. It was randomized, controlled, double-blind, parallel trial and lasted 6 months. The staest group (n=46) consumed rapeseed oil-based spread enriched with staest (3.0 g of plant stanols/d), and controls (n=46) the same spread without staest. Arterial stiffness was assessed via the cardio-ankle vascular index (CAVI) in large and as an augmentation index (AI) in peripheral arteries, and endothelial function as reactive hyperemia index (RHI). Lipids and vascular endpoints were tested using analysis of variance for repeated measurements.

**Results:**

At baseline, 28% of subjects had a normal LDL cholesterol level (≤3.0 mmol/l) and normal arterial stiffness (<8). After the intervention, in the staest group, serum total, LDL, and non-HDL cholesterol concentrations declined by 6.6, 10.2, and 10.6% compared with controls (p<0.001 for all). CAVI was unchanged in the whole study group, but in control men, CAVI tended to increase by 3.1% (p=0.06) but was unchanged in the staest men, thus the difference in the changes between groups was statistically significant (p=0.023). AI was unchanged in staest (1.96±2.47, NS) but increased by 3.30±1.83 in controls (p=0.034) i.e. the groups differed from each other (p=0.046). The reduction in LDL and non-HDL cholesterol levels achieved by staest was related to the improvement in RHI (r=−0.452, p=0.006 and −0.436, p=0.008).

**Conclusions:**

Lowering LDL and non-HDL cholesterol by 10% with staest for 6 months reduced arterial stiffness in small arteries. In subgroup analyses, staest also had a beneficial effect on arterial stiffness in large arteries in men and on endothelial function. Further research will be needed to confirm these results in different populations.

**Trial registration:**

Clinical Trials Register # NCT01315964

## Background

LDL cholesterol can be lowered with dietary means by consuming food products with supplemental plant stanol ester. Recent meta-analyses [1,2] have revealed reductions in LDL cholesterol levels by 9% at an intake of 2 g plant stanols/d, and higher daily intake resulted in enhanced LDL cholesterol reduction, up to 17% [2]. It has been estimated that each 1% reduction in the LDL cholesterol level achieves a 1% reduction in the risk of atherosclerotic coronary artery disease (CAD) [3]. Accordingly, it could be assumed that the 2 g/d dose of plant stanol consumption may reduce the risk of CAD by 9%. Unfortunately, there are no studies which have investigated whether plant stanol consumption can alter future coronary events, although the effects of plant stanols have been evaluated via surrogate indicators of cardiovascular health. Flow-mediated dilatation or brachial artery diameter has been evaluated in five short-term and one long-term study but with ambiguous results [4-9]. Arterial stiffness expressed as aortic pulse wave velocity (PWV) and endothelial function measured as pulse wave amplitude during reactive hyperemia in small arteries with peripheral arterial tonometry are novel and valid methods to assess subclinical atherosclerosis and even predict future cardiovascular events [10,11]. The cardio-ankle vascular index (CAVI) provides an assessment of arterial stiffness in large arteries reflecting the elastic properties of the arterial wall between aortic arch and lower extremities [12-14]. It has been hypothesized that CAVI might complement other techniques in the evaluation of atherosclerotic processes, e.g. endothelial dysfunction [15]. Accordingly, we thought it might represent a way to examine the effect of plant stanols on LDL cholesterol level with the working hypothesis that plant stanol consumption would have a favourable effect on cardiovascular health. The primary objective of this study was to evaluate the effects of plant stanol ester consumption on serum lipids and on surrogate indicators of cardiovascular health, i.e. arterial stiffness in large and small arteries and endothelial function.

## Methods

### Study population

Ninety-four volunteers were recruited into this study in 2011 by advertisements placed in five large companies including mainly office employees. The age range was 25–66 years with a mean of 50.8±1.0 years (SEM). Thirty-five subjects were men and 59 were women. No inclusion criteria for serum and lipoprotein lipids were set, but lipid-lowering medication or the consumption of nutrient supplements interfering with serum cholesterol level (red rice or berberine) were exclusion criteria. If the subjects had used plant sterol/stanol products, they could be included in the study after a 3 week wash out period. Other exclusion criteria were gravidity or breast feeding, unstable coronary artery disease or coronary bypass or angioplasty <6 months, inflammatory bowel disease, alcohol consumption >45 g absolute alcohol/d, or abnormal liver, kidney or thyroid function. Possible medication should have remained unchanged for 1 month before the study and, if possible, during the study.

All subjects gave their written informed consent. The study was performed according to the principles of the Declaration of Helsinki. The Ethics Committee of the Department of Medicine, Hospital District of Helsinki and Uusimaa approved the study protocol.

### Study design

The study was a randomized, placebo-controlled, double-blind, parallel clinical intervention lasting for 6 months. The 1/1 randomization was performed according to a computer generated randomization list. The plant stanol ester (staest) group consumed rapeseed-oil based spread enriched with plant stanol ester three times/d during regular meals (breakfast, lunch and dinner). The control group consumed the same spread without added plant stanols following the same instructions for frequency and timing. The subjects and the researchers were blinded to the products which were coded with computer generated different colors from Raisio Group Ltd. The color codes were only broken after all analyses had been performed.

The subjects visited the research center four times: at baseline (visit 1, randomization), and after two (visit 2), four (visit 3), and six months (visit 4, end of the study). At visits 1 and 4, blood samples were drawn after a 12-hour fast, and the vascular measurements were performed. In addition, at visits 1 and 4, a history of previous diseases, current drug treatment, use of vitamins or other nutrient supplements, and living habits were reviewed by structured questionnaires. In that questionnaire physical activity was measured asking the subjects whether they took physical exercise four times or more/week, two to three times/week, once a week, or less (physically inactive). At visits 2 and 3, the subjects collected their test margarines for the next two months, and were asked about compliance and any possible problems in the use of the test products. The subjects were contacted by telephone on three occasions: at recruitement the subjects contacted the research personnel, and their eligibility to the study was checked using a structured questionnaire. The dietitian contacted the subjects by telephone twice, during the first month after randomization and before the end of the study and checked the amounts and qualities of foods in the food records to clarify items that were unclear or missing.

### Diet

The test spreads were provided by Raisio Nutrition Ltd (Raisio, Finland). The subjects were advised to keep their habitual diet otherwise unchanged but to replace 20 g/d of their regular spread intake with the test spreads. The amount of absorbable fat excluding plant stanols was 50% in both spreads. The theoretical daily intake of plant stanols was 3 g in the staest product. The staest and control spreads contained small amounts of natural plant sterols (about 0.1 g/daily dose of spread). Compliance was verified by measuring serum plant stanols. The diet was monitored with a 3-d food record kept at baseline and at the end of the study. One of the recording days was a weekend day or the person's day off work. The nutrient intake was calculated on the Diet32 dietary analysis program (Aivo Ltd., Turku, Finland) which uses the Fineli® Food Composition Database (National Institute for Health and Welfare, Nutrition Unit, Helsinki, Finland).

### Laboratory methods and measurements

Body weight was measured with a digital scale and height with a stadiometer. Laboratory measurements (blood count, haemoglobin, serum creatinine, serum alanine aminotransferase, levels of thyroid stimulating hormone, plasma glucose, and high-sensitive C-reactive protein (hsCRP), which were taken to ensure normal health status, were analyzed with routine standardized methods at the Central Laboratory of Helsinki University Hospital (HUSLAB). Serum total, LDL, and HDL cholesterol and serum triglycerides were analyzed enzymatically using an automated analyzer systems. Non-HDL cholesterol was calculated. Serum plant stanol concentrations were quantified with capillary gas–liquid chromatography (Agilent 7890GC System, Agilent Technologies, Wilmington, DE, USA) equipped with a 50-m long Ultra 2 capillary column (5% Phenyl-methyl siloxane) (Agilent Technologies, Wilmington, DE, USA) [16] with 5α-cholestane as the internal standard.

### Vascular measurements

After 10 minutes' supine rest, blood pressure was measured manually (Boso, Germany). CAVI was measured by the analysis of PWV and pulse waveform (Vasera™ VS-1500, Fukuda Denshi Co, Japan) as described elsewhere [13].

In short, PWV is obtained by dividing vascular length by the time taken for the pulse wave to propagate from the aortic valve to the ankle. CAVI is an index of arterial stiffness reflecting the elastic properties of the arterial wall between the aortic arch and the distal arteries of the lower extremities and it is considered to be independent of blood pressure at the time of measurement [12-14,17,18]. CAVI has proven to be valid and reproducible [19,20]. In large Japanese populations, CAVI ≥8 has been found to represent increased arterial stiffness, and if CAVI ≥9, then arterial stiffness can be considered as significantly increased [21]. However, European large reference reports have not yet been published. CAVI has been proposed as a surrogate marker for athero- or arteriosclerosis [13]. It has also been used as an indicator of vascular health during dietary modification [22].

Endothelial function was assessed using peripheral arterial tonometry (PAT)(Endo-PAT2000, Software version 3.0.3, Itamar Medical Ltd, Caesarea, Israel). PAT measures pulse volume amplitude in peripheral digital arteries. The main outcome of the PAT measurement is the reactive hyperemia index (RHI), which assesses the peripheral flow induced arterial dilation after a provocation of reactive hyperemia, and it is defined as the ratio of the postdeflation pulse amplitude to the baseline pulse amplitude. This ratio is normalized to the corresponding ratio from the control arm to compensate for potential systemic changes in the amplitude. Low values of RHI reflect endothelial dysfunction. The theoretical principles behind this measurement have been described elsewhere [23]. The PAT measurement displays a good reproducibility [24].

Another outcome variable derived from the PAT measurement is the augmentation index (AI) measured in the peripheral digital arteries. This is expressed as a percentage and it reflects the stiffness of arterial system in small arteries and arterioles. PAT-derived AI is the boost increase in the late systolic pressure wave after the initial systolic shoulder [25].

### Statistical analyses

Statistical analyses were performed with SPSS for Windows 19.0 statistics program (SPSS, Chicago, IL, USA). The number of subjects recruited was based on a power analysis to detect a 10% difference in the LDL cholesterol response between the study groups with an α level of 0.05 and with statistical power of 0.80. The normality and homogeneity of variance assumptions were checked before further analyses. Univariate analysis of variance was used to compare the baseline values and the changes between the groups. The analysis of variance for repeated measurements (general linear model) was used to analyze the interaction of time and group, effects of gender, and changes over time in between-group comparisons followed by post hoc comparisons with Bonferroni corrections. For some variables of interest, Pearson or Spearman correlation coefficients were calculated. Variables not normally distributed even after logarithmic transformation, non-homogenous in variance, or non-continuous were tested with Mann–Whitney U-test, Fisher exact test, Marginal Homogeneity test, or Wilcoxon matched-paired signed rank. A p-value of <0.05 was considered statistically significant. The results are given as means±SEM.

## Results

### Baseline characteristics

The baseline characteristics of the ninety-two subjects completing the study and included in the analyses are shown in Table 1. Two subjects, one from the control and one from the staest group dropped out of the study shortly after randomization, one because of personal reasons unrelated to the study (staest group) and the other due to gastric distress (control group).

**Table 1:** Clinical characteristics, blood pressure, serum and lipoprotein lipids and vascular variables at baseline and after the six-month intervention in the control and plant stanol ester groups

	Control group (n=46)*		Staest group (n=46)*		**p**^**†**^
	**Baseline**	**Intervention**	**Baseline**	**Intervention**	
n (M/F)	46(14/32)		46(21/25)		0.197
Age (y)	50.6 ± 1.4		50.9 ± 1.4		0.676
Weight (kg) ^‡^	72.4 ± 2.0	73.3 ± 2.0	76.6 ± 2.2	77.4 ± 2.2	0.670
BMI (kg/m^2^) ^‡^	25.0 ± 0.5	25.3 ± 0.5	25.4 ± 0.6	25.6 ± 0.5	0.507
Systolic blood pressure (mmHg)^‡^	120 ± 2	118 ± 2	125 ± 2^§^	119 ± 2	0.245
Diastolic blood pressure (mmHg)	75 ± 1	74 ± 1	76 ± 1	74 ± 1	0.291
Plasma glucose (mmol/l)	4.87 ± 0.08	4.90 ± 0.08	4.97 ± 0.08	4.98 ± 0.08	0.804
hsCRP (mg/l)	1.08 ± 0.14	1.33 ± 0.24	1.01 ± 0.12	1.00 ± 0.11	0.770
Serum cholesterol (mmol/l)	5.57 ± 0.14	5.73 ± 0.15 ^ll^	5.48 ± 0.12	5.28 ± 0.11^§, ll^	<0.001
LDL cholesterol (mmol/l)	3.54 ± 0.14	3.6 ± 0.15	3.52 ± 0.12	3.23 ± 0.12^§, ll^	<0.001
HDL cholesterol (mmol/l)^‡^	1.79 ± 0.07	1.88 ± 0.07	1.76 ± 0.07	1.85 ± 0.08	0.986
non-HDL cholesterol	3.77 ± 0.15	3.85 ± 0.15	3.73 ± 0.13	3.43 ± 0.13^§, ll^	<0.001
Serum triglycerides (mmol)^‡^	0.96 ± 0.07	1.05 ± 0.07	0.89 ± 0.06	0.98 ± 0.07	0.905
CAVI cardio-ankle vascular index^111^	8.66 ± 0.16	8.73 ± 0.15	8.66 ± 0.15	8.69 ± 0.15	0.737
men (n=14/20)	8.35 ± 0.31	8.57 ± 0.29	8.55 ± 0.23	8.46 ± 0.24	
women (n=31/23)	8.81 ± 0.19	8.81 ± 0.18	8.75 ± 0.21	8.89 ± 0.18	
Reactive hyperemia index	2.2 ± 0.1	2.3 ± 0.1	2.3 ± 0.1	2.4 ± 0.1	0.610
Augmentation index, %	8.2 ± 2.5	11.5 ± 2.7^11^	8.5 ± 3.1	7.6 ± 2.5	0.046

Values shown are means±SEM. Staest, plant stanol ester; BMI=body mass index; hsCRP=high sensitive C-reactive protein.

^*^n=45/43 (control/staest group) for cardio-ankle vascular index and n=44/42 for reactive hyperemia and n=42/38 for augmentation index.

^†^Group by time interaction analyzed by repeated measures of variance analysis (general linear model),

except gender (Fisher exact test).

^‡^p<0.05, change over time.

^§^p<0.05 from controls.

^l l^p<0.05 from baseline.

^111^group by sex by time interaction p=0.024 for CAVI. In control men: baseline vs intervention p=0.061.

Fifteen subjects had hypertension, six had recovered from prostate or breast cancer, and one subject had type 2 diabetes treated by diet only (Table 2). Five subjects had a history of hypothyreosis but all were euthyreoid during the study. None of the subjects were suffering from coronary or other cardiovascular diseases. Nine of the 15 subjects with hypertension were taking regular medication. Fifteen women were receiving hormone replacement therapy, and four were using oral contraceptives or an intrauterine hormonal device. Seven subjects were smokers. The prevalences of diseases and medications were similarly distributed between the study groups as were the physical activity level, smoking, and alcohol consumption.

**Table 2:** Diseases, medication, and smoking in the control and plant stanol ester groups

	**Control group (n=46)**	**Staest group (n=46)**	**P**^**a**^
**Diseases**			
Hypertension (n)	6	9	0.574
Diabetes (n)	1	0	1
Cancer, remission (n)	2	4	0.677
Cholelithiasis (n)	2	0	0.495
Arthritis (n)	0	1	1
Celiac disease (n)	1	1	1
Hypothyreosis (n)	3	2	1
Asthma (n)	3	2	1
**Hypertension**			
Calcium channel blockers (n)	1	2	1
Beta blockers (n)	0	2	0.495
Diuretics (n)	1	1	1
Angiotensin converting enzyme- or angiotensin receptor blocking agents (n)	3	3	1
**Hormonal medication**			
Thyroxin (n)	3	3	1
Contraceptives (n)	3	1	0.623
Hormone replacement therapy (n)	8	7	1
**Smoking** (n)	5	2	0.434

^a^Difference between the groups (Fisher exact test). Staest, plant stanol ester.

The mean serum total and LDL cholesterol values were 5.5±0.1 mmol/l and 3.5±0.1 mmol/l. Sixty-six subjects (71.7%) had elevated serum total (≥ 5.0 mmol/l) and LDL cholesterol (≥3.0 mmol/l) levels. Three subjects had elevated serum triglycerides, i.e. the majority of the subjects had primary hypercholesterolemia. Almost half (47%) of the subjects were of normal weight (body mass index (BMI) ≤25 kg/m^2^) but 10% were obese (BMI >30 kg/m^2^). Systolic blood pressure was higher in the staest than in the control group (Table 1), so that the baseline systolic blood pressure value was taken as a covariate in the analyses regarding systolic blood pressure during the intervention. There were no gender-related differences between groups in terms of lipid variables and blood pressure and the nutrient intake was also similar between the groups (Table 3).

**Table 3:** Nutrient intakes during the six-month intervention

	**Control group (n=46)**		**Staest group (n=46)**		p^*^
	**Baseline**	**Intervention**	**Baseline**	**Intervention**	
Energy (MJ/d)	7.8 ± 0.3	7.4 ± 0.3	7.8 ± 0.3	7.6 ± 0.3	0.880
Fat (% of energy)	34.9 ± 1.0	35.1 ± 0.8	33.3 ± 0.9	34.8 ± 0.8	0.424
SFA (% of energy)	11.5 ± 0.5	12.1 ± 0.5	11.3 ± 0.4	11.1 ± 0.4	0.188
MUFA (% of energy)^†^	12.3 ± 0.5	12.4 ± 0.3	11.3 ± 0.4	12.8 ± 0.3	0.100
PUFA (% of energy)	5.8 ± 0.3	5.7 ± 0.3	5.4 ± 0.2	6.2 ± 0.2	0.097
Proteins (% of energy)^†^	17.1 ± 0.5	16.4 ± 0.4	17.5 ± 0.4	16.8 ± 0.5	0.886
Carbohydrates (% of energy)	40.9 ± 1	41.6 ± 0.9	42.2 ± 1.0	41.0 ± 0.9	0.168
Alcohol (% of energy)	2.6 ± 0.5	1.9 ± 0.5	2.2 ± 0.5	2.4 ± 0.5	0.169
Cholesterol (mg/d)	234.4 ± 16.7	205.2 ± 15.1	210.9 ± 11.1	212.4 ± 13.8	0.339
Cholesterol (mg/MJ)	30.5 ± 2.1	27.9 ± 1.7	27.6 ± 1.7	28.2 ± 1.6	0.358
Total fiber (g/d)	20.5 ± 1	20.0 ± 0.9	22.0 ± 1	21.6 ± 1.2	0.951
Total fiber (g/MJ)	2.7 ± 0.1	2.9 ± 0.1	2.8 ± 0.1	2.9 ± 0.1	0.615

All values are means±SEM. Staest, plant stanol ester. *SFA* Saturated fatty acids, *MUFA* Monounsaturated fatty acids, *PUFA* Polyunsaturated fatty acids. The nutrient intake at baseline did not differ significantly between the groups.

^*^Group by time interaction analyzed by repeated measures of variance analysis (general linear model).

^†^p<0.05, change over time.

CAVI was normal (<8) in 25 subjects (28%). The mean values for CAVI, RHI, and AI were similar between the groups (Table 1) nor was there any gender-related difference in these variables.

The values of CAVI correlated with age (r=0.667, p<0.001), serum total and LDL cholesterol and serum triglyceride values (r-values from 0.226 to 0.269, p<0.05), systolic blood pressure (r=0.288, p=0.008), and it tended to correlate with hsCRP (r= 0.205, p=0.055). AI values correlated with CAVI (r=0.464, p<0.001), age (r=0.499, p<0.001), BMI (r=−0.279, p=0.009) and systolic blood pressure (r=0.294, p=0.006), but not with lipids. RHI did not correlate with age, lipid variables, BMI, blood pressure, or with CAVI.

### Intervention

Weight and BMI increased in both groups similarly by 1.3±0.4% (controls) and 1.1±0.4% (staest)(p<0.05 for both) (Table 1). The clinical characteristics and all safety laboratory tests remained unchanged and no side effects were reported.

#### Feasibility of the diet

In the staest group, the serum sitostanol level was increased from 16.3±0.6 μg/dl to 30.6±1.2 μg/dl (p<0.05 from baseline and versus controls). There were no significant differences in the nutrient intakes between the groups (Table 3). The intake of monounsaturated fatty acids (MUFA) increased and the intake of protein declined similarly in both groups.

#### Serum and lipoprotein lipids

**Figure 1:**
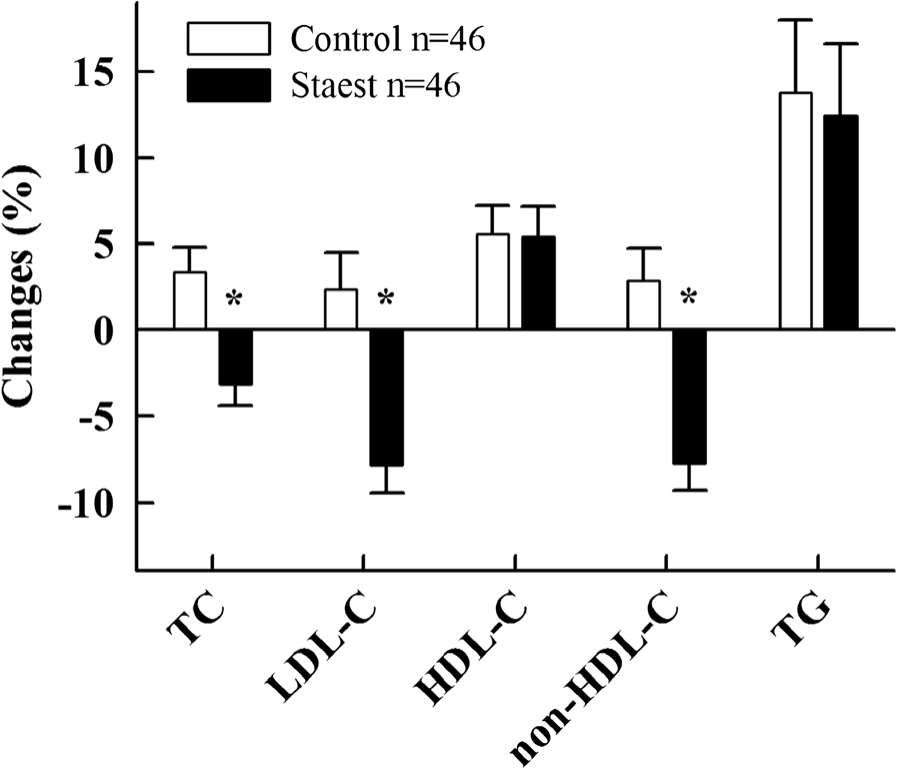
**Percent changes in serum total (TC), LDL (LDL-C), HDL (HDL-C), non-HDL cholesterol (non-HDL-C) and serum triglyceride (TG) levels in subjects consuming control and plant stanol ester (staest) spread for six months.** * p<0.05 from controls.

In the staest group, serum total and LDL cholesterol concentrations were reduced by 0.20±0.07 mmol/l and 0.29±0.05 mmol/l from baseline (p<0.05 for both) (Table 1). In the control group, serum total and LDL cholesterol levels were increased by 0.16±0.08 mmol/l (p<0.05) and 0.06±0.07 (NS). When compared with the control group, the serum total cholesterol concentration was reduced by 6.6±1.9% and LDL cholesterol by 10.2±2.7% in the staest group (p<0.001 for both) (Figure 1). Non-HDL cholesterol increased from baseline in the control group by 2.9±1.9% (NS) but was reduced by 7.8±1.5% (p<0.05) in the staest group. In comparison with the control group, staest reduced non-HDL cholesterol by 10.6±2.4% (p<0.001). HDL cholesterol and serum triglycerides were similarly increased from baseline in both groups by 5.6±1.7% (controls) and 5.4±1.8% (staest), and by 13.8±4.2% (controls) and 12.4±4.2% (staest), respectively.

#### Vascular variables

**Figure 2:**
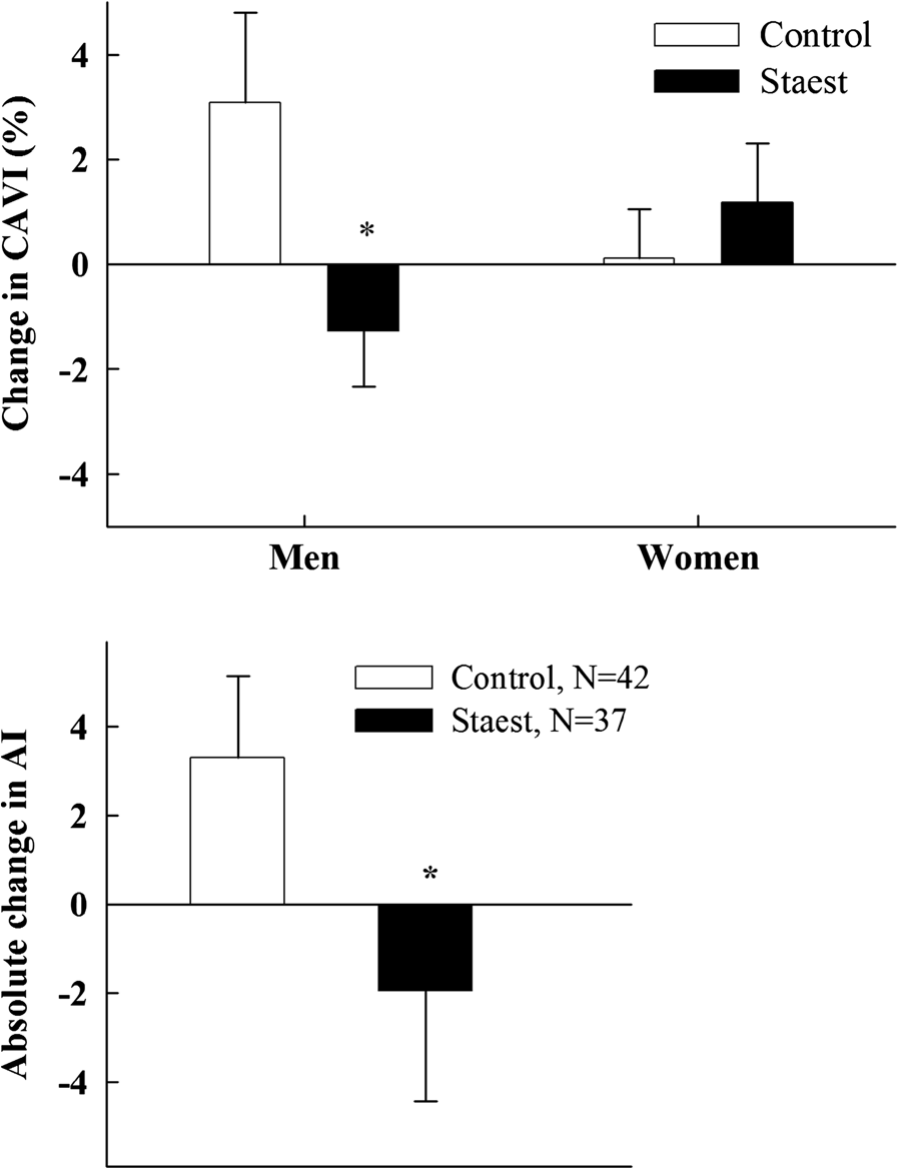
Upper panel: Percent change in cardio-ankle vascular index (CAVI) in men and women consuming control (n=45) and plant stanol ester (staest) (n=43) spread for six months. *p<0.05 from controls. lower panel: change in augmentation index (AI) in subjects consuming control and plant stanol ester (staest) spread for six months. *p<0.05 from controls.

The mean blood pressure remained unchanged during the study. In the whole population, CAVI was unchanged by staest (Table 1). However, CAVI behaved differently between men and women (Table 1, Figure 2, upper panel). In the control men, CAVI tended to increase by 0.22±0.14 (p=0.061) but was unchanged in the staest men which meant that the difference between the changes occurring during the six month trial in the two groups was statistically significant in men (p=0.023) but not in women.

The mean AI value did not change in the staest group (1.96±2.47, NS) but it increased by 3.30±1.83 in the control group (p=0.034), so that the groups differed from each other (p=0.046) (Table 1, Figure 2, lower panel).

**Figure 3:**
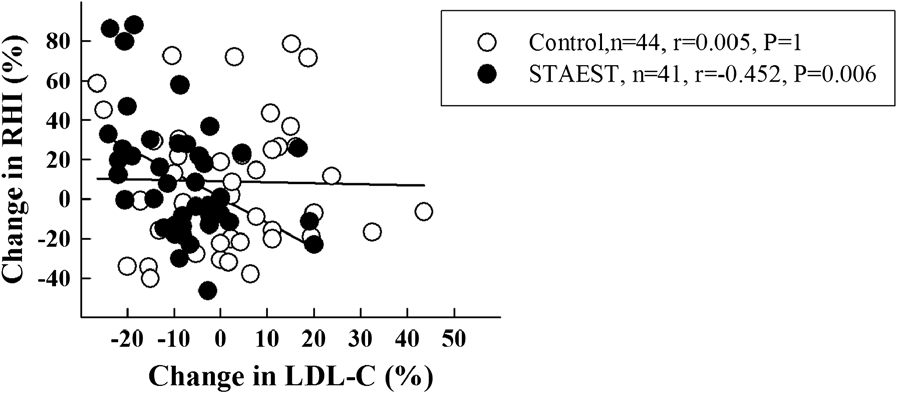
Correlation between changes (%) in reactive hyperemia index (RHI) and LDL cholesterol level in subjects consuming control and plant stanol ester (staest) spread for six months.

The mean RHI did not change significantly in either group (Table 1). However, in the staest group the change in LDL cholesterol level was related to the change in RHI (Figure 3). A similar association was observed between the changes in the levels of non-HDL cholesterol and RHI in the staest group (r=−0.436, p=0.008). The changes in vascular variables did not differ in subjects responding (n=39) or not responding (n=7) to LDL cholesterol lowering with plant stanol ester.

## Discussion

The novel finding in this study was that consumption of plant stanol esters for 6 months reduced arterial stiffness in small arteries (AI). In large arteries (CAVI) in men, the plant stanol ester supplement prevented the progression of arterial stiffness observed in the control men. Furthermore, endothelial function was improved by plant stanol ester in relation to the reduction in LDL and non-HDL cholesterol.

The study population was recruited from commercial companies and was mainly office employees; there were no inclusion or exclusion criteria for lipid values. The reason for unfixed lipid criteria was that we wanted to recruit a cohort representing as far as possible the general adult population without lipid lowering medication. Seventy-two % of the subjects had elevated LDL cholesterol level (>3.0 mmol/l) and in this respect resembled the adult Finnish population [26]. The dietary intake of fats, especially those of saturated fatty acids was too high according to the dietary recommendations for hypercholesterolemia (National Cholesterol Education Program 2002. Available at: http://www.nhlbi.nih.gov/guidelines/cholesterol/). During the intervention, the consumption of the rapeseed-oil based spread ameliorated the fatty acid intake by increasing the intake of MUFA.

In the staest group, the serum sitostanol concentration was increased by 96%, which is the same magnitude as observed in our previous studies [7,27] and is indicative of good compliance.The control-related 10% reduction in LDL cholesterol value was within the range described in previous plant stanol studies [2].

With regard to the arterial stiffness in large arteries, it seemed that 28% of the study population had normal values at baseline (CAVI<8), and thus may have affected the results of the intervention. CAVI was related to age, blood pressure, serum total and LDL cholesterol and serum triglyceride levels, and AI. The change in LDL cholesterol due to the consumption of plant stanol esters was not associated with the change in CAVI, which is in line with the results of a previous study with eicosapentaenoic acid [28]. At baseline, there was no gender difference in CAVI in contrast to an earlier finding [13], but during this intervention the response in CAVI was sex-related which is a novel observation. Since CAVI tended to increase in the controls but remained unchanged in the staest men, it can be postulated that consumption of the plant stanol ester had prevented the progression of arterial stiffness in large arteries during the 6 months of this intervention.

CAVI has been studied earlier in three lipid intervention studies [28-30]. In all these studies, the intervention was based on one change, i.e. intake of pitavastatin, ezetimibe or eicosapentaenoic acid, with all other parameters including dietary and lifestyle habits left as unchanged as possible in free-living subjects. The interventions lasted from three months up to one year. In the only placebo-controlled study, 1.8 g/day of eicosapentaenoic acid for 3 months reduced CAVI in 92 subjects with the metabolic syndrome by 3.6% in conjunction with reducing serum concentrations of triglycerides, C-reactive protein and serum amyloid A in LDL, and an increased serum adiponectin level [28]. In the two open, uncontrolled studies with type 2 diabetic subjects, CAVI was significantly decreased by 6.6% after 1-year of pitavastatin treatment [29], and by 1.9% after a 6- months’ trial with ezetimibe [30]. In these studies, the changes in CAVI were not large even though significant, and in the two latter studies the reduction in CAVI was dependent on its high baseline value. Accordingly, one possible reason for the nonsignificant change in CAVI in the study population receiving staest in the present study might be the fact that one-third of the subjects had normal CAVI values at baseline. As a whole, CAVI is a novel technique to assess arterial stiffness. Although there are large-scale promising results about the use of CAVI in some non-Caucasian populations, further studies will be needed to clarify its usefulness in different populations [31].

The reductions in the levels of LDL and non-HDL cholesterol over the 6 months resulted in reduced arterial stiffness in small arteries. No previous studies have investigated the relationship between plant stanols and AI, and in fact few studies have evaluated the effect of LDL cholesterol lowering on AI derived from the PAT signal. In a recent study, consumption of omega-3 fatty acids, 4 g/d for 16 weeks had no effect on AI [32]. Statin treatment has been demonstrated to exert a beneficial effect on AI as measured with other techniques [33-35]. Further studies will be needed to assess the usefulness of AI as an additive parameter in the evaluation of endothelial function with the PAT technique. The present results suggest that it might be easier to improve arterial stiffness in the peripheral small arteries and arterioles than in the large arteries. It has been claimed that improving blood flow in small arteries, arterioles and even in microcirculation could have a major clinical relevance [36].

It seemed that endothelial function as assessed with RHI was improved in post hoc analyses only, similarly to our previous study [5]. The reductions in LDL cholesterol and non-HDL cholesterol were inversely associated with the change in RHI suggesting that the more one could reduce LDL and non-HDL cholesterol levels, the more RHI would be improved. Since over two-thirds of the subjects were hypercholesterolemic at baseline, one could speculate that their endothelial function was impaired; however, the mean RHI values were not low [11], which may have affected the result.

## Conclusions

Lowering LDL and non-HDL cholesterol levels by 10% with plant stanol ester consumption for 6 months reduced arterial stiffness in small arteries in a symptomless cohort of adults with varying levels of LDL cholesterol and markers of subclinical atherosclerosis. The subgroup analyses revealed that consumption of plant stanol esters exerted a beneficial effect also on endothelial function and on arterial stiffness in large arteries in men. Further investigations will be needed to confirm these results in different populations.

## Abbreviations

AI: Augmentation index; BMI: Body mass index; CAVI: Cardio-ankle vascular index; hsCRP: High-sensitive C-reactive protein; MUFA: Monounsaturated fatty acids; PAT: Peripheral arterial tonometry; PUFA: Polyunsaturated fatty acids; PWV: Pulse wave velocity; RHI: Reactive hyperemia index; SFA: Saturated fatty acids; Staest: Plant stanol ester.

## Competing interests

The authors declare that they have no competing interests. Raisio Nutrition Ltd supported the study with a grant and provided the test products, but had no role in the design and performing the study or in the contents of the manuscript.

## Authors’ contributions

HG, HL, MH, AS, and AT were all responsible for the study design. HG, HL, MH, JH, JK, PS, MJN, AS and AT carried out the study. PS was responsible for collection of all data except the dietary data, MH was responsible for the collection of dietary data and performed the statistical analyses. HG drafted the first version of the manuscript, which was revised and completed by JH, HL, JK, PS, MJN, AS, AT and MH. All authors read and approved the final manuscript.

## Pre-publication history

The pre-publication history for this paper can be accessed here:

http://www.biomedcentral.com/1471-2261/13/50/prepub
